# Obesity in Qatar: A Case-Control Study on the Identification of Associated Risk Factors

**DOI:** 10.3390/diagnostics10110883

**Published:** 2020-10-29

**Authors:** Md. Tawkat Islam Khondaker, Junaed Younus Khan, Mahmoud Ahmed Refaee, Nady El Hajj, M. Sohel Rahman, Tanvir Alam

**Affiliations:** 1 Department of Computer Science and Engineering, Bangladesh University of Engineering and Technology, Dhaka 1205, Bangladesh; 1405036.mtik@ugrad.cse.buet.ac.bd (M.T.I.K.); 1405051.jyk@ugrad.cse.buet.ac.bd (J.Y.K.); msrahman@cse.buet.ac.bd (M.S.R.); 2 College of Science and Engineering, Hamad Bin Khalifa University, Doha 34110, Qatar; mrefaee@hbku.edu.qa; 3 Geriatric Department, Hamad Medical Corporation, Doha 3050, Qatar; 4 Faculty of Medicine, Ain Shams University, Alabasia 38, Cairo, Egypt; 5 College of Health and Life Sciences, Hamad Bin Khalifa University, Doha 34110, Qatar; nelhajj@hbku.edu.qa

**Keywords:** obesity, overweight, BMI, machine learning, bone mineral composition, bone mineral density, Qatar, Qatar Biobank (QBB)

## Abstract

Obesity is an emerging public health problem in the Western world as well as in the Gulf region. Qatar, a tiny wealthy county, is among the top-ranked obese countries with a high obesity rate among its population. Compared to Qatar’s severity of this health crisis, only a limited number of studies focused on the systematic identification of potential risk factors using multimodal datasets. This study aims to develop machine learning (ML) models to distinguish healthy from obese individuals and reveal potential risk factors associated with obesity in Qatar. We designed a case-control study focused on 500 Qatari subjects, comprising 250 obese and 250 healthy individuals- the later forming the control group. We obtained the most extensive collection of clinical measurements for the Qatari population from the Qatar Biobank (QBB) repertoire, including (i) Physio-clinical Biomarkers, (ii) Spirometry, (iii) VICORDER, (iv) DXA scan composition, and (v) DXA scan densitometry readings. We developed several machine learning (ML) models to distinguish healthy from obese individuals and applied multiple feature selection techniques to identify potential risk factors associated with obesity. The proposed ML model achieved over 90% accuracy, thereby outperforming the existing state of the art models. The outcome from the ablation study on multimodal clinical datasets revealed physio-clinical measurements as the most influential risk factors in distinguishing healthy versus obese subjects. Furthermore, multiple feature ranking techniques confirmed known obesity risk factors (c-peptide, insulin, albumin, uric acid) and identified potential risk factors linked to obesity-related comorbidities such as diabetes (e.g., HbA1c, glucose), liver function (e.g., alkaline phosphatase, gamma-glutamyl transferase), lipid profile (e.g., triglyceride, low density lipoprotein cholesterol, high density lipoprotein cholesterol), etc. Most of the DXA measurements (e.g., bone area, bone mineral composition, bone mineral density, etc.) were significantly (*p*-value < 0.05) higher in the obese group. Overall, the net effect of hypothesized protective factors of obesity on bone mass seems to have surpassed the hypothesized harmful factors. All the identified factors warrant further investigation in a clinical setup to understand their role in obesity.

## 1. Introduction

Obesity is a chronic, multifactorial disease associated with multiple comorbidities including diabetes, cardiovascular disease, stroke, hypertension as well as different types of cancers [1,2,3]. Diabetes has the strongest association with obesity [4], where more than 80% of type 2 diabetes cases are either overweight or obese [5]. Furthermore, high BMI levels are associated with colon and thyroid cancer in men, gallbladder and endometrial cancer in women, and renal and esophageal cancer in both genders [6]. Obesity is also considered as one of the risk factors for osteoarthritis [7,8,9], ischemic stroke [10], and atrial fibrillation [11]. Moreover, lipoprotein abnormalities, such as a change in high-density lipoprotein cholesterol, are closely related to obesity [12]. 

Obesity and its related comorbidities have a negative impact on the health care systems in several countries. In this regard, Qatar is not an exception as reported by Mandeya et al. [13], where the negative impact of obesity on the community, healthcare services, and economy of the country is described. In the last three decades, Qatar witnessed a significant increase in the number of overweight and obese individuals [13]. In 2006, the World Health Survey (WHS) reported that 16% of Qatari children were overweight based on BMI levels set by the World Health Organization (WHO) [13]. Subsequently, in 2012, following the guidelines of the WHO STEPwise approach to Surveillance (STEPS), the national survey reported ~ 70% of the Qatari population to be overweight (BMI > 25 kg/m^2^), and 41% to be obese (BMI > 30 kg/m^2^) [14]. As a result, the rate of obesity-related comorbidities is relatively high in Qatar. Informatively, this rate is comparable to the neighboring Gulf countries [14]. Therefore, identifying obesity risk factors can help to improve the health and well-being of obese subjects as well as support individuals suffering from obesity-related comorbidities.

Previous studies ([15,16,17,18]) used a limited number of physio-clinical biomarkers to model obesity and its related risk factors. There are a few studies that have considered socio-economic factors, cultural impact, dietary habits, and psychological status from the Qatari population to determine how such factors might lead to obesity [13,19,20,21]. Nevertheless, no study to date has used a large variety of multimodal physio-clinical measurements from obese subjects enrolled in the Qatar Biobank (QBB). The only study to employ a machine learning (ML) approach to identify obesity risk factors in the QBB cohort revealed albumin, uric acid, insulin, and c-peptide as potential risk factors for obesity in Qatar [22]. The model described in [22] was developed on 60 clinical measurements and achieved ~82% accuracy in separating the obese group from the control group. The objective of our study is to develop a new ML model to distinguish obese individuals based on 236 clinical measurements, collected from QBB [23,24] and to identify obesity-associated risk factors in the Qatari population. In the sequel, we tested the hypothesis that known risk factors for obesity are applicable to the Qatari population and whether novel risk factors specific to the Qatari population exist. To the best of our knowledge, this is the first study to apply ML models on a multimodal dataset of anthropometric measurements, arterial stiffness, respiratory function, bone mineral density, bone mineral composition, etc., to determine potential obesity risk factors in the Qatari population. 

## 2. Methods 

### 2.1. Ethical Approval

This study was conducted under the regulation of the Ministry of Public Health, Qatar. All procedures were approved on 2 May 2020 by the Institutional Review Board (IRB) of Hamad Medical Corporation, Qatar for Ex -2019-RES-ACC-0164-0087, and only de-identified data were collected from QBB.

### 2.2. Cohort Description

We collected data from the QBB, one of the largest biobank projects in the middle east [23,24]. QBB is a national population-based cohort study that collects data and biomedical samples from the adult (aged above 18 years) Qatari population. Consented participants were invited to visit the QBB premise, and they were interviewed by medical practitioners to collect and record their previous health and family history. Then, each of the participants went through an extensive physical and clinical examination. The details of the data collection protocol are described in [23,24]. Out of all enrolled participants, 250 obese (cases) and 250 control individuals were selected randomly with the help of QBB medical practitioners as part of this case-control study. The obese group comprised adult participants (aged above 18 years) with BMI ≥ 30 (kg/m^2^), who were free from diabetes, cardiovascular disease, and cancer. As controls, we selected a group of non-obese individuals with no history of diabetes, cardiovascular disease, stroke, sleep disorder, hypertension, or cancer. All the subjects from the studied cohort were Qatari nationals. 

### 2.3. Physio-Clinical Measurements 

The Omron 705 device (Omron Corporation) [25] was used to capture two systolic and diastolic BP measurements. If the readings differed by ≥5 mmHg, another measurement was taken. Seca Bio Impedance Analyzer (Seca GmbH & Co. KG, Hamburg, Germany) was employed to obtain the BI measurements for the participants in QBB. Additionally, Seca Stadiometer was used to capture anthropometric measurements (e.g., weight, height, waist circumference, hip circumference, etc.) Blood samples were collected to measure different clinical biomarkers representing bone and joint function, coagulation test, diabetes, full blood count, white cell count, steroid hormones, lipid profile, liver function, thyroid functions, vitamins, etc. The respiratory function for each of the participants was assessed based on Spirometry using the Pneumotrac Vitalograph (Vitalograph (Ireland) Ltd., Ennis, Ireland) [26]. VICORDER device (SMT medical GmbH & Co. KG; Bristol, UK) was used to assess the arterial stiffness of the participants [27]. In QBB, total body BMD value (gm/cm^2^) was obtained using dual-energy X-ray absorptiometry (DXA, General Electric Company, Madison, Wisconsin, USA) scan for each participant. Bone densities at forearms, spine, and femur sites were obtained and total body BMD was measured for the entire body [28]. The details of the data collection protocol at QBB can be found in [23,24]. In total, 236 measurements were collected for each participant and considered as features for the ML models. Among these features, 70 were based on the physio-clinical biomarkers, 5 were collected from the VICORDER device, 35 were related to Spirometry, and 126 were measured using the DXA machine. Table 1 summarizes the feature categories including a few examples from each category. 

### 2.4. Data Pre-Processing

Obese (normal) subjects were considered as the positive (negative) class in our classification model. The available dataset contained a small number of missing values (<0.01%); therefore, we discarded any feature with >20 missing values; otherwise, we replaced each missing value by the mean value of corresponding measurements in the same class. 

### 2.5. Feature Subset Selection

Feature selection is an important step for any classification task where the goal is to select only features that are rich in discriminatory information with respect to the classification problem at hand. We leveraged the so-called *filtering* approach (as opposed to a wrapper approach) where we select features as a preprocessing step, independent of the choice of the actual classification/learning algorithm. The idea is to exploit the information included in the dataset (e.g., the correlation between variables and discriminatory abilities of the individual features) to create the most promising feature subset by discarding irrelevant ones before the commencement of learning. 

In an attempt to filter out uncorrelated features, we used two different feature subset selection (FSS) techniques to identify a subset of features (see Supplementary File 1). First, we applied the FSS technique based on PCC to determine the correlation matrix. Then, we filtered-out strongly correlated features if their absolute *p*-value is below a certain threshold (1 × 10^−60^) (namely, weight, waist, and hip size). In this method, we kept the features achieving the highest absolute PCC-value with bioimpedance Z-FMI value (as mentioned in “Dataset Pre-Processing” section) and discarded the remaining features which are obvious to determine obesity. We considered a correlation threshold that indicates the maximum allowable absolute correlation between any pair of features. For any pair of features exceeding this threshold, we retained the feature having the higher absolute PCC-value with bioimpedance Z-FMI and discarded the remaining feature. We considered the correlation threshold as a hyperparameter and tuned it from 0.3 to 0.9. Additionally, we used Gini index [29] to determine the relative importance of each individual feature. We selected the total feature number as another hyperparameter and considered the Gini index as threshold. Next, we filtered-out the features with a lower Gini index relative to this threshold. In our experiments, we tuned the total feature number to 10, 15, 20, 25, 30, 35, 40, 45 and 50. 

### 2.6. Statistical Significance of the Variables Selected by Feature Subset Selection Techniques

We used the Anderson–Darling test [30] to check whether the variables are normally distributed. For normally distributed variables, we used the student’s t-test [31] to determine the significance level for each variable (*p*-value < 0.05) when comparing the obese versus the control group. For other variables, we applied a Mann–Whitney [32] test for the same purpose.

### 2.7. Machine Learning Model Development

**Figure 1:**
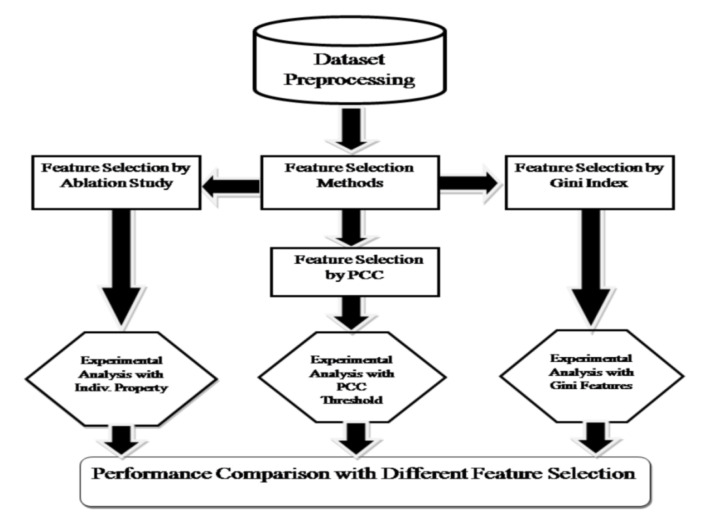
Workflow of feature selection methods and their performance comparison using machine learning (ML) models.

For the development of our ML model, we first discarded certain measurements from our analysis that are expected to have sufficient distinguishing power to separate the obese group from the control group, e.g., BMI, weight, Z-FMI, hip and waist circumference, and waist to hip ratio. The clinical measurements described in Table 1 were only considered in our model. We tested six ML algorithms: linear support vector machine (SVM) [33], SVM with radial basis function (RBF) kernel [34], decision tree [35], naïve Bayes [36], random forest (RF) [29], and gradient boosting (GB) [37]. We set the penalty parameter at 1.0 for RBF SVM, and we used 10,000 estimators for the random forest. For gradient boosting, we used 100 estimators and set the learning rate to 0.1. Figure 1 summarizes the computational workflow used in this study to assess the importance of different measurements based on various classification models.

### 2.8. Model Evaluation

The training and validation were performed using a 10-fold cross validation (CV) on 90% of the available data, and the remaining 10% were used for independent testing. To analyze the performance of different ML models, the following performance evaluation metrics were used (Equations (1)–(4)): (i) precision, (ii) recall, (iii) accuracy, and (iv) Matthews Correlation Coefficient (MCC).

(1) $$Accuracy=\frac{TP+TN}{TP+TN+FP+FN}$$

(2) $$Sensitivity=\frac{TP}{TP+FN}$$

(3) $$Precision=\frac{TP}{TP+FP}$$

(4) $$MCC=\frac{TP*TN-FP*FN}{\sqrt{\left(TP+\;FP\right)\left(TP+FN\right)\left(TN+FP\right)\left(TN+FN\right)}}$$

Here, TP, FN, FP, and TN stand for true positive, false negative, false positive, and true negative, respectively. 

## 3. Results

The studied cohort included an equal number of males and females in each of the obese and control group. The average age for the subjects in the obese and control group were 35.89 years and 30.28 years, respectively (Table 2). When we stratified the participants based on age, we found 116 (23%), 208 (42%), 122 (24%), 54 (11%) participants in the age groups 18–25, 26–35, 36–45, and 46–64, respectively. The average BMI value for the obese group was 34.60 kg/m^2^ whereas the control group had an average BMI of 23.29 kg/m^2^. Table 2 further highlights baseline characteristics in both groups. BMI and Bioimpedance Z-score of fat mass index (Z-FMI) measurements were not included in the feature vector for building our ML model since they are obvious factors associated with obesity. We found that both Z-FMI and BMI are highly correlated (correlation = 0.975; *p*-values close to 1 × 10^−83^) where modeling with any of these two variables naturally exhibits near perfect accuracy. We compared the correlation between Z-FMI and other numerical features and subsequently discarded features with a very low Pearson Correlation Coefficient (PCC) (i.e., score within a range of (−0.05, 0.05)). In the subsequent analysis, a total of 57 features with low PCC were discarded (Supplementary File 2).

### 3.1. Performance of Machine Learning Models Based on Ablation Study

Here, we performed an ablation study using different types of features (see Table 1) as well as their combinations to distinguish the obese from the control group. We observed that a GB based model achieved the best performance after all types of features were combined (Table 3). On the other hand, physio-clinical biomarkers and DXA composition properties achieved >80% accuracy. The best precision (0.881), recall (0.891), and MCC (0.763) were achieved by selecting all features together.

### 3.2. Performance of ML Models Considering the Selected Feature Subset 

**Figure 2:**
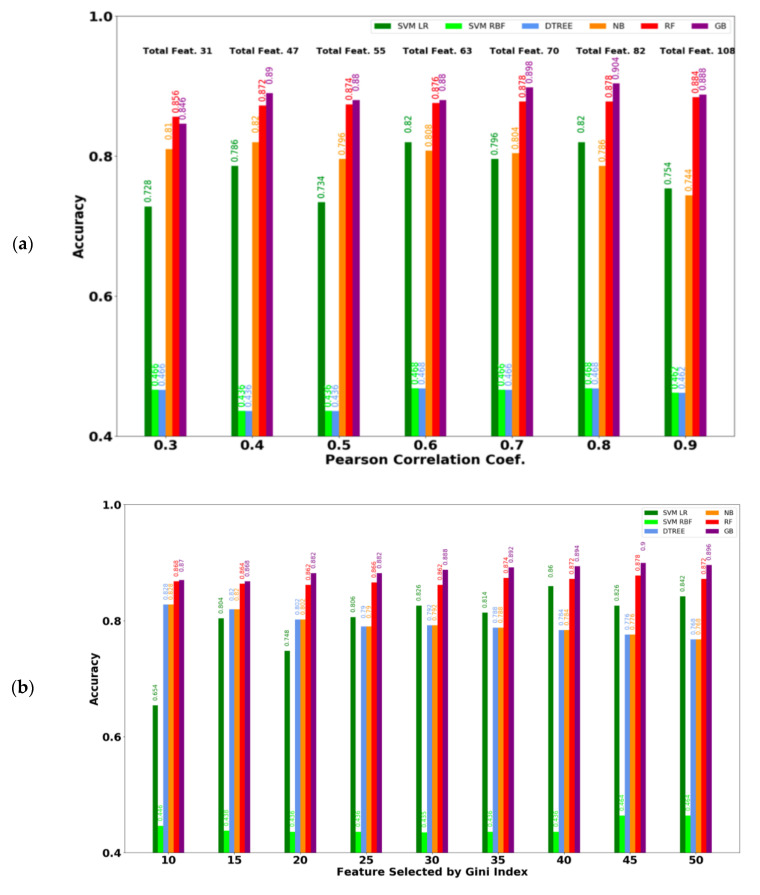
Accuracy comparison of different ML models with two feature selection methods. (**a**) Features are selected by Pearson Correlation Coefficient (PCC). Best accuracy (0.904) is achieved by Gradient Boosting with 82 features when the maximum allowable PCC threshold between any two features is 0.8. (**b**) Features are selected by Gini index. Best accuracy (0.90) is achieved by Gradient Boosting with the top 45 features.

Figure 2a shows the performance for different PCC thresholds with the best result for each metric displayed in bold. This analysis revealed that the best accuracy (0.904), precision (0.908) and MCC (0.806) were achieved using the 0.8 threshold containing 82 features. The best recall (0.896) was achieved for the 0.4 threshold containing 47 features. Figure 2b shows the performance of various features selected according to the Gini index, where the best result for each metric is highlighted in bold. The best accuracy (0.9), precision (0.912), and MCC (0.798) were achieved using the top 45 Gini important features, whereas the best recall (0.891) was achieved when the top 50 features were considered. The proposed ML models achieved more than 90% accuracy in distinguishing the obese group from the control group thereby outperforming the existing model [22], which achieved 82% accuracy for the same purpose. Supplementary File 1 summarizes all the selected features by both FSS techniques as well as their corresponding statistics.

### 3.3. Performance of ML Models on Gender and Age Based Stratified Participants 

Supplementary File 3 highlights the performance of ML models when the dataset is stratified according to age and gender. We observed that the GB-based model achieved the best accuracy for an age range of 18–45 years. On the other hand, linear-SVM achieved the best accuracy for an age range of 46–64 years. We also found that the best accuracy for age range 46–64 was 0.83, and the best MCC was 0.498. Whereas, the best accuracy and the best MCC for the other age ranges were close to 0.87 and 0.7, respectively. This can be due to the low number of participants (54) for age range 46-64 when compared to other age groups. As a result, the models could not learn due to the limited number of data. Moreover, the best accuracy (0.931) for the age range 18–25 and the best accuracy (0.9) for the age range 36–45 were higher than the best accuracy (0.882) for the combined ages (Table 3). Therefore, it can be deduced that the features we used to identify obesity showed more efficacy for age ranges 18–25 and 36–45. For gender-based stratification, the GB-based model achieved the best accuracy (0.9) for both male and female classes. More importantly, the performances of each model on male and female classes are almost equal, which indicates that the selected features for obesity detection were unbiased to gender.

### 3.4. Statistical Significance of the Variables Selected by Feature Subset Selection Techniques

**Figure 3:**
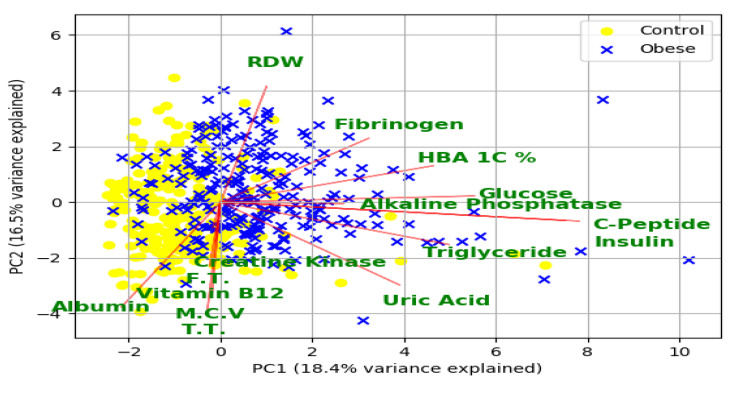
Principal component analysis (PCA) biplot for obesity based on the top 15 biomarkers selected by feature subset selection (FSS) techniques. T.T.: Testosterone Total, F.T. = Free Triiodothyronine, M.C.V.= Mean Cell Volume.

Supplementary File 1 highlights the list of statistically significant features (97 features) that were selected by feature selection methods (PCC and Gini index-based methods). As evident from this list, 35 of the selected features are related to DXA, 42 related to physio-clinical biomarkers, five related to VICORDER reading, eight related to Spirometry measurements, and the remaining measurements were obtained from physiological and demographic information. Figure 3 shows the principal component analysis (PCA) biplot of the first two principal components based on the top 15 clinical biomarkers measured by the Gini index and selected by PCC as well. The graph indicates overlapping clusters of control and obese cases detected by the first two principal components. These two principal components can explain almost 35% of the variance from these 15 biomarkers. The vectors for the biomarkers indicate a high correlation between c-peptide and insulin and also among free triiodothyronine, testosterone total, vitamin B12, mean cell volume, and creatine kinase. The direction of fibrinogen, red blood cell distribution width (RDW), and HbA1c% is almost opposite to the direction of albumin, free triiodothyronine, and creatine kinase. This opposite direction indicates that these biomarkers are inversely correlated to obesity, which is justified by the corresponding PCC values of the biomarkers (Supplementary File 1).

### 3.5. Potential Risk Factors for Obesity and Related Morbidities 

Our model identified 42 potential biomarkers that require further in-depth analyses to be considered as obesity risk factors (Supplementary File 1). Among these biomarkers, 34 are statistically significant (*p*-value < 0.05) when comparing the obese vs. control group. C-peptide, insulin, glucose, HbA1c, uric acid, fibrinogen, free triiodothyronine, albumin, gamma glutamyl transferase (GGT), and alkaline phosphatase are the top ten ranked biomarkers for obesity (based on *p*-value). This list also includes four known risk factors (c-peptide, insulin, albumin, and uric acid) for obesity, previously reported in [22].

### 3.6. Bone Mineral Density Associated Factors in Obesity

We found 35 DXA measurements from six different categories to be significantly different between the obese and control group (Supplementary File 1). These variables represent (a) bone Area, (b) bone mineral composition (BMC), (c) bone mineral density (BMD), (d) Z-score, (e) percent of age matched, and (f) average height/weight in bone marrow. The mean value of most DXA measurements were significantly higher (*p*-value < 0.05) in the obese group. 

## 4. Discussion

### 4.1. Principal Findings

In this study, we leveraged the potential of ML for two main purposes. First, we developed a classification model to check if the bio-clinical measurements are sufficient to distinguish the obese group from the control group with high accuracy. The second purpose was to identify important bio-clinical measurements that can be considered as potential risk factors for obesity. This study differs from other ML based works from the literature (e.g., [15,16,17,18]) along two important lines. Firstly, previous works consider BMI as one of the features; however, we excluded this feature since it is an obvious determinant of obesity and ensures a near perfect accuracy. Secondly, previous works consider only a limited number of physio-clinical biomarkers whereas our research includes a comprehensive set thereof making our study unique in this context. Additionally, application of FSS techniques provided the relative importance of the clinical biomarkers that were used in this study (Supplementary File 1).

From clinical biomarkers, we observed that HbA1c, Glucose, Insulin, and c-peptide are higher in the obese vs. control group (Supplementary File 1). This indicates the higher incidence of diabetes in obese individuals, which aligns with the results of several large-scale epidemiological studies [38,39]. In our study, uric acid (UA) was observed as highly prevalent in the obese group. Epidemiological studies have shown a link between serum uric acid (UA) and increased rates of cardiovascular events. Hyperuricemia occurs frequently in obese subjects as well as hypertensive patients. Furthermore, elevated levels of serum uric acid are strongly associated with metabolic syndrome [40,41]. Obesity is also known to affect liver function through various mechanisms. We found that fatty liver, synthetic liver functions like albumin, and total protein are lower in the obese group (Supplementary File 1) whereas different liver enzymes (e.g., alkaline phosphatase, gamma glutamyl transferase (GGT)) are higher in the obese group. We believe that these changes can be potentially attributed to steatosis [42], which indicate that obese people are more prone to liver disorders compared to non-obese individuals. For lipid profile related markers, obesity is well known to go hand in hand with dyslipidemia. Here, we noticed that triglyceride and low-density lipoprotein (LDL) cholesterol are significantly higher in the obese group whereas high density lipoprotein (HDL) cholesterol is significantly lower therein. These markers suggest that the obese population has a higher risk of cardiovascular complications (Supplementary File 1). Although thyroid-stimulating hormone (TSH) was not measured in our study, serum TSH was previously reported to be positively correlated with BMI in a cross-sectional study of 736 euthyroid adults [43]. In our study, we observed that free thyroxine and free triiodothyronine are significantly lower in the obese group (Supplementary File 1). Lower levels of free thyroxine and free triiodothyronine may play a role in the development of obesity [44]. Furthermore, we detected slightly higher creatinine levels in the obese group compared to the control group (Supplementary File 1). Although creatinine is a non-specific biomarker of kidney function, several studies have found obesity as one of the risk factors for de novo chronic kidney disease [45]. Obesity can play an indirect role in the development of chronic kidney disease via increasing hypertension and diabetes mellitus, as well as through an increase in inflammatory processes and interleukin. We also observed that both systolic BP and diastolic BP are significantly higher in the obese group compared to the normal group (Supplementary File 1). Higher BP is a well-known risk factor for heart diseases; therefore, the obese group is at higher risk for several BP related comorbidities such as heart failure and stroke.

Salamat et al. [46] previously showed that obesity was highly associated with bone mineral density of the hip and lumbar spine. We also found that the distribution for L1 width, area of L4, BMC, and age matched BMD of L2 are significantly higher in the obese group as compared to the control group (Supplementary File 1). Moreover, we observed that obesity and increased weight are associated with higher BMC and BMD in most body parts for the obese group. These findings can be explained by processes reviewed in López-Gómez et al. [47], where the different actions of obesity on bone health and metabolism are summarized. Even though the association between obesity and bone metabolism is still controversial [48], and increased body weight may have a positive impact on bone health [49,50]. The positive effect of obesity on bone health can be due to an increase in bone mass in response to a mechanical load on the bone [47]. We found that BMD in several areas, including arms, head, neck, ribs, spine, trunk, and troch are significantly higher in the obese group (Supplementary File 1). Additionally, an increase in fat mass is associated with higher androgen to estrogen conversion, which is reflected by a positive stimulus for bone metabolism. These results consolidate the occurrence of lower androgen levels in the obese group that might be further converted to estrogen. We further identified a lower level of testosterone in the obese group (average level of 9.25 nmol/L) when compared to the control group (average level of 11.33 nmol/L), which matches the result reported by Kirschner et al. [51]. Insulin plays a pivotal role in postnatal bone growth as well as the function of bone cells. The current study shows a significantly higher level of insulin in obese individuals that might provide additional bone protection. Moreover, bone formation can be stimulated by amylin secretion secondary to insulin resistance by beta cells. On the other hand, it is important to emphasize that obesity may have a negative impact on bone fracture [52]. The factors that may have an adverse effect on bone metabolism are pro-inflammatory states associated with the secretion of a number of cytokines (IL-6, TNF-) and adipocytokines (adiponectin, leptin, vitamin D). Obese patients have been previously shown to have decreased circulating levels of 25-hydroxy vitamin D [47]. This is in line with our study where we detected slightly lower vitamin D in the obese (average level of 15.54 ng/mL) versus control group (average level of 15.59 ng/mL). This decrease in vitamin D levels can be attributed to vitamin sequestration in adipose tissue of obese individuals. Based on our findings, the net effect of hypothesized protective factors of obesity on bone mass may have surpassed the hypothesized harmful factors. 

### 4.2. Limitations

One of the limitations of our study is that the average age in the obese (35.89 years) group is 5 years higher than the control (30.28 years) group in this Qatari cohort. Since the average age was below 40 years in both groups, we believe that age-related changes in obesity disparity between the 2 groups are minimal. One additional limitation is that this study was conducted on a relatively small number of participants within the context of ML. This limits the use of deep learning architectures, which could have led to a better performance overall. Nevertheless, the ease of model interpretability as well as the 90% accuracy level using traditional ML models have encouraged us to continue relying on these models for further analyses. In the future, we will try to extend our work on a larger cohort with the support from QBB. 

## 5. Conclusions

In this study, we integrate a wide variety of multimodal clinical datasets to develop highly accurate ML models to distinguish obese from non-obese subjects. Our ML models achieve more than 90% accuracy thereby outperforming the existing state of the art model. The proposed ML-based model confirms the previously reported risk factors (e.g., c-peptide, insulin, albumin, uric acid) for obesity and suggests additional biomarkers pertinent to morbidities such as diabetes (e.g., HbA1c, glucose), liver function (e.g., alkaline phosphatase, gamma-glutamyl transferase), bone-joint function, lipid profile, etc. Interestingly, we observe a net positive impact of the hypothesized protective factors of obesity on bone mass (e.g., bone area, bone mineral composition, bone mineral density, etc.) as compared to the hypothesized harmful factors. The advantage of the proposed ML based system is its ability to relate the diagnosis to the associated risk factors that may not be obvious with a plain eye in a clinical setup. Our ML based approach could act as a “support system” for the physicians along with the existing clinical decision support systems (CDSS) to provide access to a trusted, reliable source that would help determine a better treatment plan for obese individuals. We could foresee that the proposed ML model would be used in reducing the risk of future comorbidities in obese individuals. Additionally, such a data-driven ML based system, if established in a clinical setup with proper validation, would reduce the time and cost spent on patient treatment by improving healthcare management for patients and physicians. We believe that our results would contribute to future research endeavors in this direction.

## Figures and Tables

**Table 1:** Summary of available measurements used in this study.

Features	Size	Examples
All	236	Spirometry, Physio-clinical Biomarkers, VICORDER, DXA
Spirometry	35	Forced expiratory time (FET), Forced expiratory flow (FEF), Forced Vital Capacity (FVC), etc.
Physio-clinical Biomarkers	70	Hemoglobin, red blood cells, white blood cells, Lymphocyte, Platelet count, Sodium, Urea, Glucose, Cholesterol, Systolic blood pressure, Diastolic blood pressure, hip waist circumference, etc.
VICORDER	5	Heart Beats, Heart rate, PPI (Pulse Pressure Index), Pulse Wave (PWV)
DXA body composition	5	CT bone mass android, CT bone mass android visceral, CT bone mass arms, CT bone mass gynoid, CT bone mass total
DXA Densitometry	121	DT area arms, DT area head, DT area pelvis, DT area spine, DT area total, etc.

**Table 2:** Baseline characteristics for the obese and the control groups.

		Obese		Control		
	Unit	Mean	Standard Deviation	Mean	Standard Deviation	*p*-Value
Age	year	35.89	9.898	30.28	8.32	1.54 × 10^−11^
BMI	kg/m^2^	34.60	4.08	23.29	2.81	1.10 × 10^−83^
Weight	kg	94.22	15.15	63.99	10.74	9.38 × 10^−72^
Z-FMI	-	2.65	1.13	−0.24	0.708	1.99 × 10^−83^
Waist circumference	cm	97.45	10.71	75.02	8.58	8.85 × 10^−94^
Hip circumference	cm	116.66	8.80	97.16	6.75	2.30 × 10^−77^
waist-to-hip ratio (WHR)	-	0.84	0.087	0.77	0.076	5.27 × 10^−16^

**Table 3:** Performance of the models based on ablation study ^1^.

Property	Evaluation Parameter	SVM (linear)	SVM (rbf)	Decision Tree	Naïve Bayes	RF	GB
**All**	Accuracy	0.806	0.47	0.812	0.688	0.868	**0.882**
	Precision	0.874	0.305	0.803	0.701	0.85	**0.881**
	Recall	0.748	0.58	0.813	0.678	**0.891**	0.879
	MCC	0.635	0.068	0.616	0.379	0.739	**0.763**
**Spirometry**	Accuracy	0.543	0.518	0.557	0.543	0.587	**0.628**
	Precision	0.354	0.578	0.557	0.522	0.582	**0.618**
	Recall	0.313	0.146	0.521	**0.750**	0.578	0.611
	MCC	0.045	0.12	0.112	0.099	0.178	**0.256**
**VICORDER**	Accuracy	0.525	0.565	0.583	0.593	0.633	**0.64**
	Precision	0.597	0.545	0.579	**0.746**	0.635	0.645
	Recall	0.441	0.574	0.587	0.246	**0.603**	**0.603**
	MCC	0.090	0.126	0.17	0.23	0.266	**0.28**
**Physio-clinical Biomarker**	Accuracy	0.679	0.489	0.645	0.75	0.802	**0.807**
	Precision	0.729	0.04	0.644	0.767	0.814	**0.821**
	Recall	0.657	0.1	0.634	0.692	0.772	**0.786**
	MCC	0.408	0.0	0.293	0.502	0.609	**0.621**
**DXA Body Composition**	Accuracy	0.754	0.438	0.792	0.736	**0.832**	0.83
	Precision	0.833	0.269	0.794	0.755	0.84	**0.841**
	Recall	0.717	0.6	0.799	0.697	**0.823**	0.817
	MCC	0.545	0.012	0.581	0.473	**0.665**	0.661
**DXA Densitometry**	Accuracy	0.68	0.436	0.682	0.626	0.758	**0.79**
	Precision	**0.834**	0.27	0.690	0.643	0.734	0.785
	Recall	0.565	0.6	0.661	0.597	**0.813**	0.803
	MCC	0.422	0.0	0.368	0.26	0.523	**0.586**

^1^ The numbers highlighted in bold represent the highest value for the corresponding evaluation metric.

## Supplementary Materials

The following are available online at https://www.mdpi.com/2075-4418/10/11/883/s1, File 1: List of features selected by FSS techniques along with their corresponding statistical significance, File 2: List of discarded features along with their corresponding statistics, File 3: Performance of machine learning models on gender and age stratified participants.
