# Supplementary material for: Obesity in Qatar: A Case-Control Study on the Identification of Associated Risk Factors
Source: Diagnostics (Basel). 2020 Oct 29;10(11):883. doi: 10.3390/diagnostics10110883 (PMC7693222; doi:10.3390/diagnostics10110883)
Supplement: Supplementary file 1 [file diagnostics-10-00883-s001.zip › SupplementaryFiles/Additional-File-3.docx]

***Performance of ML models following gender-based stratification:***

**Table**: Performance of the models for males and females.

| **Gender** | **Evaluation Parameter** | **SVM (linear)** | **Decision tree** | **Naïve Bayes** | **RF** | **GB** |
| --- | --- | --- | --- | --- | --- | --- |
| **Male** | Accuracy | 0.868 | 0.816 | 0.695 | 0.88 | **0.9** |
|  | Precision | **0.927** | 0.81 | 0.701 | 0.856 | 0.916 |
|  | Recall | 0.809 | 0.82 | 0.69 | **0.915** | 0.881 |
|  | MCC | 0.751 | 0.631 | 0.396 | 0.759 | **0.795** |
| **Female** | Accuracy | 0.869 | 0.837 | 0.749 | 0.869 | **0.909** |
|  | Precision | 0.879 | 0.823 | 0.734 | 0.884 | **0.938** |
|  | Recall | **0.891** | 0.884 | 0.791 | 0.863 | 0.888 |
|  | MCC | 0.759 | 0.675 | 0.504 | 0.748 | **0.824** |

***Performance of ML models following age-based stratification:***

**Table :** Performance of the models for different age groups.

| **Age** | **Evaluation Parameter** | **SVM (linear)** | **Decision tree** | **Naïve Bayes** | **RF** | **GB** |
| --- | --- | --- | --- | --- | --- | --- |
| **18-25**  **(116 participants)** | Accuracy | 0.897 | 0.879 | 0.761 | 0.906 | **0.931** |
|  | Precision | 0.887 | 0.8 | 0.623 | 0.843 | **0.917** |
|  | Recall | 0.847 | **0.879** | 0.702 | 0.83 | 0.863 |
|  | MCC | 0.791 | 0.748 | 0.475 | 0.768 | **0.838** |
| **26-35**  **(208 participants)** | Accuracy | 0.75 | 0.74 | 0.639 | 0.812 | **0.842** |
|  | Precision | 0.79 | 0.727 | 0.624 | 0.801 | **0.837** |
|  | Recall | 0.78 | 0.727 | 0.638 | 0.806 | **0.83** |
|  | MCC | 0.566 | 0.479 | 0.284 | 0.626 | **0.69** |
| **36-45**  **(122 participants)** | Accuracy | 0.736 | 0.737 | 0.697 | 0.819 | **0.9** |
|  | Precision | 0.873 | 0.793 | 0.909 | 0.791 | **0.921** |
|  | Recall | 0.739 | 0.788 | 0.615 | 0.97 | 0.931 |
|  | MCC | 0.487 | 0.312 | 0.429 | 0.573 | **0.714** |
| **46-64**  **(54 participants)** | Accuracy | **0.83** | 0.663 | 0.787 | 0.783 | 0.763 |
|  | Precision | **0.877** | 0.753 | **0.877** | 0.797 | 0.81 |
|  | Recall | 0.897 | 0.757 | 0.847 | 0.98 | 0.897 |
|  | MCC | **0.498** | 0.17 | 0.47 | 0.245 | 0.326 |
